# Micro- and Nanoscale Hydrogel Systems for Drug Delivery and Tissue Engineering

**DOI:** 10.3390/ma2020577

**Published:** 2009-05-13

**Authors:** Christine T. Schwall, Ipsita A. Banerjee

**Affiliations:** Department of Chemistry, Fordham University / 441 East Fordham Road, New York 10458, USA; E-Mail: schwall@fordham.edu (C.T.S.)

**Keywords:** hydrogel, micro, nano, drug delivery, tissue engineering, cancer therapy, diabetes, biosensor

## Abstract

The pursuit for targeted drug delivery systems has led to the development of highly improved biomaterials with enhanced biocompatibility and biodegradability properties. Micro- and nanoscale components of hydrogels prepared from both natural and artificial components have been gaining significant importance due to their potential uses in cell based therapies, tissue engineering, liquid micro-lenses, cancer therapy, and drug delivery. In this review some of the recent methodologies used in the preparation of a number of synthetic hydrogels such as poly(*N*-isopropylacrylamide) (pNIPAm), poly(ethylene glycol) (PEG), poly(ethylene oxide) (PEO), polyvinyl alcohol methylacrylate co-polymers (PVA-MA) and polylactic acid (PLA), as well as some of the natural hydrogels and their applications have been discussed in detail.

## 1. Introduction

The quest for targeted drug delivery systems has led to the development of highly improved biomaterials with enhanced biocompatibility and biodegradability properties. Such drug delivery systems can be made of natural components such as chitosan, gelatin, polysaccharides and silk fibroin or synthetic materials such as carbon nanotubes, silica nanostructures, triblock copolymer gelators, synthetic hydroxyapatite beads, and polyelectrolyte microcapsules [1,2,3,4,5,6,7,8,9,10,11]. Many researchers have chemically modified these materials to further enhance their efficacy and biocompatibility. For example, due to their low toxicity and the fact they are also stable in acidic environments acetal-derivatized dextrans have been prepared for incorporation into materials that are both hydrophilic and hydrophobic [12]. Other beneficial drug delivery vehicles that have been studied include polyketal (PK) microparticles, which may overcome the critical problems of polyester and polyanhydride drug delivery systems that suffer from polyphasic drug release profiles. This problem may be compounded if the target area is in an acidic environment, or characterized by a high rate of clearance such as tumors, inflammations, and phagosomes [13]. PK particles contain ketal linkages in their backbone, which allows significant flexibility in their design. Such particles have several advantages, such as longevity, and can be engineered in a variety of sizes, shapes, and porosities and can degrade over different periods of time, ranging from 1-2 days to weeks at a variety of pH values. Further, they can break down into non-acidic compounds such as acetone and diols, which are innocuous to resident proteins [14,15].

A common methodology used to synthesize drugs targeted to specific tissues or organs is to combine the drug of interest with a ligand that can bind to a receptor on the cell surface. For example, researchers have conjugated ribavirin with hemoglobin for treatment of hepatitis C [16]. In another study, monomethylauristatin E (MMAE)-albumin was conjugated with RGD-peptides [17,18]. RGD-peptide sequences, which are recognized by the integrin cell surface receptor and play a key role in cell-adhesion, have also been conjugated to poly-(ethylene oxide)-*block*-poly(ε-caprolactone) (PEO-*b*-PCL) micelles, with the prospect that these vehicles can be used to enhance the adhesion and uptake of drug-loaded particles in the metastatic tumor’s blood vessels and cancer cells [19].

The use of transferrin-gold nanoparticles as ligands, liposomal doxorubicin (DOX) for interaction with folate receptors, and the application of magnetite- encapsulated micro- and nano-scale polymer particles have also been widely experimented [20,21,22]. Cell-specific cytokines or antibodies, liposomes, and synthetic polymers can also be utilized as ligands to which the drugs can be bound [23]. Further, dendrimer-based micelles are incredibly beneficial with regard to targeted drug delivery in cancer treatment because the micelles are pH and temperature sensitive and they can be connected to ligands specific for cancerous cells [24].

Amongst the several types of drug delivery systems that have been developed in order to improve effectiveness and biocompatibility, hydrogels are extremely promising. Hydrogels are biocompatible hydrophilic networks that can be constructed from both synthetic and natural materials. Some examples include photocrosslinkable polymers, poly(amidoamines), poly(l-lactide)-poly(ethylene oxide)-poly(l-lactide)(PLA-PEO-PLA), gellan gum, hyaluronic acid, and calcium alginate [25,26,27,28,29,30,31,32,33,34,35]. The gels are able to swell due to the large amount of water they can hold and can also undergo changes in shape or volume in response to physical or biological conditions such as temperature, pH, ionic concentration, or specific antigens [36,37]. Hydrogels have tremendous potential in various applications due to their injectability, relatively low cytotoxicity, biodegradability, mucoadhesiveness and tunable bioadhesive properties. These properties make hydrogels highly attractive materials for tissue regeneration and for drug delivery to specific sites in the body [38]. The benefits of injectable gels also include softness, ease of manipulation, high water content, and nontoxicity [39,40,41]. This review presents an overview of the various synthetic methods used to synthesize hydrogels, and their applications specifically in the development of highly biocompatible materials, which can be utilized for a number of potential biomedical applications. 

Some common materials used to synthesize hydrogels include poly(*N*-isopropylacrylamide), poly(ethylene glycol), poly(l-lactic-co-glycolic acid), methacrylated poly(glycerol succinic acid) dendrimers, and hen egg white lysozyme [42,43]. In the case of hen egg lysozymes, thermo-reversible hydrogels were formed in the presence of a reducing agent such as dithiothreitol. The resulting hydrogels obtained were found to be highly biocompatible with fibroblasts and could potentially be utilized as cell scaffolds [44,45]. Protein-*graft*-PEG hydrogels have also displayed cell adhesiveness and mechanical strength, which is promising particularly with respect to applications in tissue regeneration [46]. Photo-cross-linkable oligo[poly(ethylene glycol) fumarate] (OPF) hydrogels have been used to encapsulate chondrocytes, and the materials can then be injected into the body for usage in cartilaginous tissue engineering [47].

In recent times, the importance of size scale of hydrogels used has become increasingly evident. Thus, micro- and nanoscale hydrogels have become highly popular due to their improved potential uses in cell-based therapies, tissue engineering, liquid microlenses, and drug delivery systems [48,49]. They are particularly useful because they can reach areas of the body not accessible to macroscale hydrogels and enter the cytoplasm of cells. Further, they also have a large surface area that can be used as a conjugation surface to specialize the hydrogels for specific targets [50]. Nanoscale hydrogels can have extended circulation times, making them an ideal material for drug delivery systems for the eye as well [51,52,53,54].

## 2. Synthetic Polymer Based Hydrogels

### 2.1. Poly(N-isopropylacrylamide) in Drug Delivery and Biosensing

Recently, Hoare and Pelton have polymerized poly (*N-*isopropylacrylamide) (pNIPAm)-based microgels using acrylic acid (AA), methacrylic acid (MAA), vinylacetic acid (VAA), or fumaric acid (FA) to alter the carboxylic acid content that is functionalized in the gel. The VAA and FA functionalized gels were found to have functional groups mostly located on the microgel surface, while AA had a relatively uniform distribution of functional groups throughout the gel. MAA on the other hand was found to have functional groups within the core of the gel [55]. The uptake and release of various drugs, of varying charges such as dibucaine, desipramine (cationic drugs), dopamine, acetaminophen (neutral) and naproxen (anionic), within the functionalized microgels was then studied. The researchers found that the core-functionalized gels took up cationic drugs more efficiently because they tended to bind to the carboxyl groups. 

**Figure 1:**
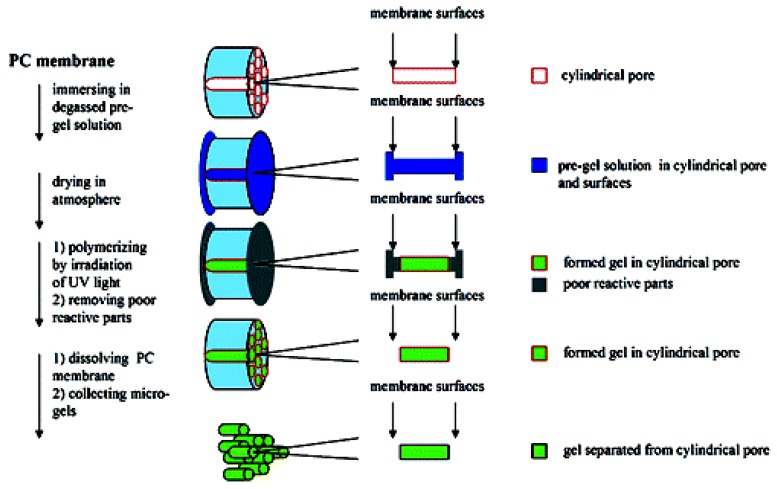
Template-guided synthesis combined with photopolymerization for the development of pNIPAm-based cylindrical microgels. [58], *Copyright*, *Reprinted with permission from the American Chemical Society*.

Techniques to develop an optimally sized nano pNIPAm gel with maximal volume change to be utilized in drug delivery systems have also been studied [56,57]. Recently, Tsujii and co-workers have succeeded in making cylindrical pNIPAm-based microgels by an approach in which template-guided synthesis and photochemical polymerization methods were combined [58]. It was found that by using this combination method, pNIPAm microgels of uniform sizes could be developed, with the size of these gel cylinders showing consistency with the pores within the polycarbonate membrane used as the template. A schematic for the formation of the pNIPAm microgels using this method is shown in Figure 1. This technique is promising for the development of these microgels and they can be more readily available for drug loading and delivery.

**Figure 2:**
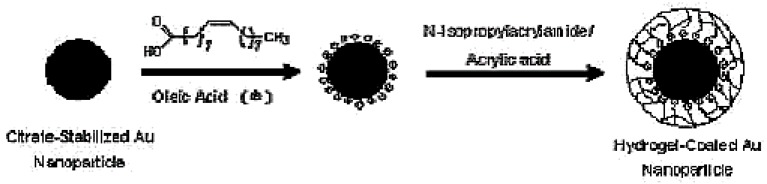
Schematic for the Synthesis of hydrogels on the surface of gold nanoparticles using surfactant-free emulsion polymerization (SFEP). [60], *Copyright*, *Reprinted with permission from the American Chemical Society*.

pNIPAm was also copolymerized with acrylamide (Am) to form core-shell microcapsules that were loaded with gold nanoparticles (AuNPs) through a liquid-liquid dispersion technique [59]. Microwave, photo and thermo-responsive polymer microgels in a size range of 500 to 800 μm swollen with water were then prepared by microarray technique. The water-in-oil droplets containing 26 wt% pNIPAm and Am monomers, 0.1 wt% Tween-80 surfactant, FITC fluorescent dye and the colloidal gold nano-particles spontaneously formed a core-shell morphology that was fixed by *in situ* photopolymerization. The gels showed reversible swelling and deswelling. Further, the AuNP/pNIPAm hybrid core-shell microcapsules and microgels could be actuated by visible light and/or microwave radiation or temperature. Such microgels may have potential applications in microfluidic switches or microactuators, photosensors, and nanomedicinal applications in controlled drug delivery and release. The core of the microgels embedded with nanoparticles can hold a liquid that can be released over a range of temperatures and radiations indicating that this material may have implications in controlled and targeted drug delivery. Lee and co-workers studied the structural and optical properties of hybrid nanoparticles made of a small gold core coated with a biocompatible hydrogel shell consisting of the biocompatible thermo-responsive copolymer derived from a mixture of pNIPAm and acrylic acid [60]. The thickness of the shell was varied between 20 and 90 nm. The nanoparticles were developed as drug-delivery vehicles that were responsive to both temperature and pH changes. Further, those hydrogel nanoparticles could be thermally activated by exposure to light. Their work demonstrated that such hybrid materials for drug delivery could be prepared through surfactant-free emulsion polymerization as shown in Figure 2. Recently, Lyon and co-workers have explored the formation of pNIPAm nanogels by growing pNIPAm shells onto metal nanoparticle seeds [61]. It was found that when the adsorbed pNIPAm layer, was heated above the lower critical solution temperature, the pNIPAm layer collapsed onto the Au nanoparticle surface. The pNIPAm layer served as a hydrophobic nucleus for growing pNIPAm oligoradicals, resulting in the formation of a pNIPAm shells. Etching of the Au core from the polymer-coated particles resulted in hollow hydrogel nanoparticles. Such hydrogel-coated nanoparticles have tremendous potential as drug carriers. 

In another study, micro-, and nanoscale hydrogels were synthesized from pNIPAm-*co*-(1-vinyl-imidazole) (pNIPAm-VI). Kazakov and co-workers demonstrated the concept of hydrogel ionic reservoirs for hydrogel particles of three different size ranges, namely macrogels (1,000 − 6,000 μm), microgel (~ 20 − 200 μm), and nanogels (~ 0.2 μm) [62]. The ion sensitivity of pNIPAm-VI microgels with imidazolyl (ionizable) groups was confirmed by the pH dependence. Their results indicated that diffusion of ions inside and outside the gel and the binding to ionizable groups along the polymer network occurred with a higher efficiency for hydrogel particles, which were smaller in size. The authors proposed a two-step mechanism to illustrate the changes in the proton concentration in the solution external to hydrogel particles. The first step involved a rapid binding of ions to the immediate surface of each particle, and second, a successive diffusion of bound ions into the next inner layer of the polymer network. Further, it was assumed that the ions from exterior would bind to the newly vacant sites on the particle surface.

The method applied in their work can be useful for characterization of artificial systems such as drug delivery vehicles, actuators, biosensors, wet nanoelectronics and for natural structures such as cytoplasm, cells, mitochondria and bacterial spores. 

**Figure 3:**
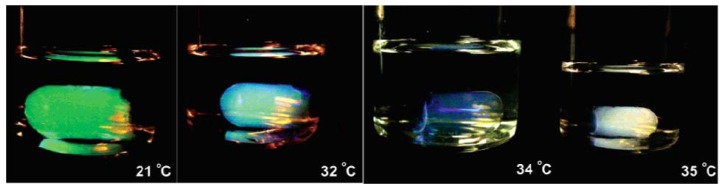
pNIPAm-*co*-allylamine crystal hydrogels switching colors with the change of the temperatures. From left to right, the temperatures are 21, 32, 34, and 35 °C. [63], *Copyright*, *Reprinted with permission from the American Chemical Society*.

Using precipitation polymerization method, pNIPAm was cross-linked with allylamine to form pNIPAm-*co*-allylamine microgels in the presence of *N,N*’-methylenebisacrylamide (BIS) as a cross-linker [63]. In order to synthesize these microgel particles into a crystalline structure, the dispersions of pNIPAm-*co*-allylamine microgels were ultracentrifuged, heated and then cooled. The resulting crystalline structures were covalently cross-linked with glutaric dialdehyde. Results indicated that at higher cross-linking density of the gel, the colloidal crystals form at higher polymer concentrations. Further, the hydrogels with higher polymer concentrations have a higher mechanical strength, at physiological pH, which is important in potential use as drug delivery systems and biomedical applications. The crystalline hydrogels at room temperature displayed a bright green color and as the temperature was increased, the color of the gel changed from green to blue at 31 °C and to milky white at 35 °C, just at the volume phase transition temperature of the particles (as shown in figure 3). When the temperature was decreased to room temperature again, the gel restored its color and volume. The process was completely reversible. In addition, Bragg diffraction wavelength changes were monitored using UV-vis spectroscopy and it was found that for the microgel dispersion, shrinkage of the gel due to temperature changes does not alter the Bragg diffraction wavelength, while an increase in the pH from 7 to 12, decreases the Bragg diffraction peak. These results indicate that these microgel colloidal dispersions may also be used as sensors in detecting changes in environmental conditions.

Hydrogel nanoparticles co-polymerized by aqueous, free-radical, precipitation polymerization of pNIPAm with acrylamide using the cross-linking agent BIS also formed ordered crystalline gels [64]. It was found that the crystals shift between ordered and disordered states depending on the thermal conditions of the environment. At higher temperatures, the crystals were less ordered and the solution could be manipulated while still allowing an ordered recrystallization to occur if the temperature was lowered. The results of this study hold promise in forming thin-film materials that could be utilized to construct environmentally responsive nanomaterials that may be utilized in biosensing or in constructing functional superstructures.

Poly(acrylamide) (pAm), pNIPAm, and pNIPAm-VI, were used to prepare nanogels within liposomes [65]. Large unilamellar vesicles (LMV) derived from l-α-phosphatyidylcholine were prepared in chloroform, followed by addition of the cross-linker, and photoinitiator. In all cases, the reactions were carried out in the presence of *N,N’*-diethoxy-4,4’-azobis(pyridinium) hexafluoro-phosphate (DEAP) as photoinitiator and methylene bisacrylamide (MBA) was selected as the tetrafunctional crosslinker. After photopolymerization was completed, the lipid bilayer was dissolved using detergent, and the lipid components were then removed so that the formed nanogels could be dried. *N*-(*n*-octadecyl) acrylamide (ODAm), which is hydrophobic, was also attached to the surface of the pNIPAm-VI hydrogel nanoparticles, forming lipobeads, in order to allow the nanoparticles to be attached to the surface of lipid bilayers. The results illustrated by their work indicate that these lipobeads undergo reversible swelling and de-swelling indicating that the aggregates formed were not fusing together when the volume changes occurred. Further, compared to the other nanogels, the pNIPAm-VI lipobeads had a higher stability over temperature and pH changes. This could be due to the fact that changes in the gel size are hard to observe since the hydrophobic outer layer is highly stable. Overall these experiments hold promise in studying ionic reservoirs, sensory systems, and controlled release systems because of the mechanical stability of the lipid molecule surrounding the nanogel.

#### 2.1.1. pNIPAm Hydrogels for Drug delivery in Treatment for Diabetes

The polymer pNIPAm has also been used to form microgels in which insulin has been impregnated [66]. A previously developed method, where in a dried polymer gel was allowed to re-hydrate in a solution of the desired material that needed to be loaded, was utilized to encompass the insulin within the network of the hydrogel [67,68]. It was found that this “breathing-in” technique is better at encapsulating the insulin when compared to more traditional encapsulation methods in which the gel is already swelled when put into contact with the material to be loaded. Further, stages of insulin release were found to be a function of thermal changes, which is important to note if the pNIPAm gels are used in future drug delivery system. In a separate study, core–shell microgels with degradable pNIPAm as the core and non-degradable phenylboronic acid (PBA)-conjugated poly(*N-*isopropylacrylamide) [p(NIPAm-PBA)] as the shell were synthesized [69]. It was found that at room temperature, the degraded polymer segments diffused freely out of the precursor poly(*N*-isopropylacrylamide-co-acrylic acid) gel shells in water. In contrast, the PBA-modified p(NIPAm-PBA) nanoshell could hold most of the degraded core polymer chains under the same conditions, due to its condensed structure at the collapsed state. Lowering the temperature or increasing pH increased the swelling degree of the p(NIPAm-PBA) shell, which provides methods to control its permeability by temperature and pH. The complexation of PBA groups with glucose also enhanced the swelling of the nanoshell and, increased its permeability. The understanding of how to control the permeability of the glucose-sensitive nanoshell in hollow microgel particles is very important for the design of self-regulated insulin delivery systems. Researchers have also polymerized gels using pNIPAm as a base, which is composed of various concentrations of MAA, AA, fumaric acid (FA), and vinylacetic acid (VAA), and 3-aminophenylboronic acid (APBA) as a functional component because phenylboronic acids have been noted to interact with carbohydrates [70]. It was found that these gels responded to changes in environmental glucose concentration, with the APBA functional groups controlling the magnitude of the swelling induced by the glucose. It was also discovered that the concentration of the varying polymers comprising the pNIPAm base as well as the amount of APBA copolymerized onto the base of the gel controls the amount of swelling or de-swelling that occurs over a certain range of glucose concentrations.

#### 2.1.2. pNIPAm Hydrogels as Actuators 

Capsules made up of colloidal (hollow capsule) microgel spheres also hold promise in being used as microscopic actuators because they are able to respond to various stimuli. A precipitation polymerization procedure was used to synthesize the microgel particles from pNIPAm-*co*-AA and then the colloidosomes were produced by adding an aqueous pNIPAm-*co*-AA solution to 2-octanol, which formed droplets stabilized with the pNIPAm-*co*-AA [71]. The structures were further stabilized by locking the microgel particles together through addition of poly(butadiene-*b-N*-methyl-4-vinyl pyridinium iodide) to the octanol. When the droplets were dissolved and the microparticles transferred to an aqueous solution, stable colloidosomes were formed. These colloidal microgels responded to changes in temperature and it was found that as the temperature of the surrounding environment increased, the microgel spheres shrank in size, but the change in size was only reversible between 28.0 °C and 42.5 °C. The results show promise in developing microscopic actuators or pumps that may have use in the body. Other materials that may have implications in developing actuators are microgel thin films. An aqueous free-radical precipitation polymerization process was used to synthesize microgels from pNIPAm and AA with BIS as a cross-linker. Then a spin-coating layer-by-layer (scLbL) technique was used to assemble the thin films from the previously synthesized microgels and poly(allylamine hydrochloride) (PAH) [72]. The results revealed that depending on whether the microgel or the PAH forms the top layer, the film can act as a bi-material or homogenous film depending on the pH conditions of the environment. When the microgel composed the top layer of the film, it responded to changes in pH in a similar way to particles in free solution, but the film has also been seen to behave in such a way that reflected increased cross-linking underneath particle layers when the pH was increased. These results reflect the ability of these films to be used in developing soft actuators.

#### 2.1.3. pNIPAm Hydrogels in Ocular and Lens Applications

Microsystems based on hydrogels are also being used in developing liquid lens systems that are capable of independently focusing [73,74,75]. The hydrogel polymers used in the manufacture of soft contact lenses are usually hydrophilic materials and hence expected to have a highly wettable surface, when fully hydrated. The surface wettability of hydrogel materials may be influenced by the tendency of the materials to lose water progressively through dehydration and the intrinsic mobility of the polymer chains that allows rotation of hydrophilic groups away from the hydrogel surface when in contact with more hydrophobic interfaces such as by deposition of lipids [76]. In general, the surface tension of a hydrogel material gradually increases with water content up to a certain level when it is in equilibrium with water. However, when in contact with hydrophobic interfaces the surface tension decreases rendering the material less wettable. Thus all conventional hydrogel materials can be expected to have adequate wettability when fully hydrated. The basic strategy developed by Jiang and co-workers for making microsystem lenses involves a hydrogel ring that is capable of responding to stimuli embedded within a microfluidic channel system contained between a glass plate and an aperture slip [77]. When exposed to various stimuli, the hydrogel ring underneath the aperture opening in the microsystem expands or shrinks, altering the volume and curvature of the water droplet within the ring, which changes the angle at which light is able to enter the system. PNIPAm, AA, and 2-(dimethylamino)ethyl MA (DMAEMA) hydrogels were used. Two types of smart liquid microlenses were fabricated. The first was the temperature-sensitive pNIPAm hydrogel that expanded at low temperatures and contracted at high temperatures. The volume transition temperature was found to be approximately 32 ºC. In the second type, pH-sensitive hydrogels using AA hydrogel, which expanded in basic solutions and contracted under acidic conditions were prepared. The benefits of such microlenses, regardless of the hydrogel material used, include the ability for autonomous response, and the option to integrate it within existing electronic systems in order to develop the lens by *in situ* liquid-phase photopolymerization. In another study, a series of other hybrid polymeric hydrogels, prepared by the reaction of acrylic acid-functionalized chitosan with either pNIPAm or 2-hydroxyethyl methacrylate monomers, were synthesized, and investigated for their ability to act as controlled release vehicles for ophthalmic drug delivery [78]. The effects of network structure and composition upon the swelling properties, adhesion behavior, and drug release characteristics were investigated.

### 2.2. Polyethylene glycol (PEG) Based Hydrogels in Biosensing, and Drug Delivery

**Figure 4:**
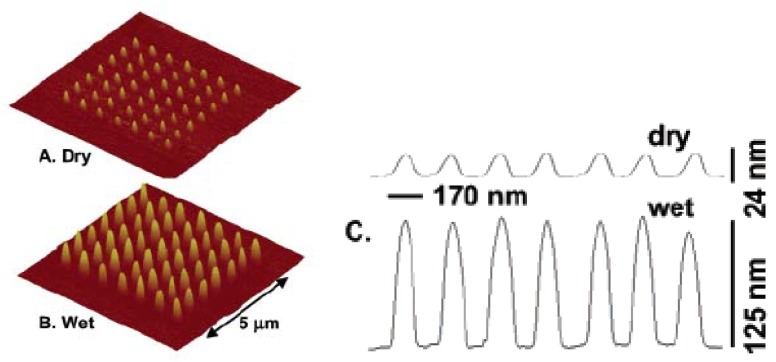
AFM images of a 5 μm × 5 μm array of amine-terminated PEG 5000 nanohydrogels with an inter-gel spacing of 715 nm. (A) Dry; (B) Hydrated; (C) height profiles of a row of nanogels with swell by a factor exceeding five times. [83], Copyright, *Reprinted with permission from the American Chemical Society.*

Nanoscale PEG hydrogels can also be utilized as substrates to produce multifunctional surfaces that may have applicability in bio- and proteomic sensors [79,80,81,82]. For example, monoamine-terminated PEG 5,000 (PEG-NH_2_) thin films were cross-linked on silicon substrates (as shown in Figure 4) using a focused electron beam that allows substantial amounts of energy to be targeted to very specific locations. In order to show that the same gel could be functionalized with different proteins, including laminin and fibronectin, bovine serum albumin (BSA)-amplified PEG-NH_2_ nanogels were constructed using the photoactivatable heterobifunctional cross-linker, sulfosuccinimidyl-6-[4’-azido-2’-nitrophenylamino] hexanoate (sulfo-SANPAH). Results revealed that the gels could be made multifunctional, which is important in designing protein chips that can be utilized as biosensors [83]. 

Another method developed to synthesize nanoscale hydrogels integrating PEG, involved the use of liposomes as templates to synthesize hydrogel nanoparticles consisting of PEG-diacrylate (PEG-DA) via photopolymerization. Once the hydrogels were synthesized, the lipid component was removed by dissolving it in choloroform. The nanoparticles were then functionalized with aldehyde groups to allow them to interact with materials containing amine functional groups [84]. In the next step, β-galactosidase enzymes were encapsulated within the hydrogel nanoparticles while the nanoparticles were being constructed and no loss of the enzyme activity was observed. Such hydrogel nanoparticles have potential use in specific drug delivery and drug targeting systems. Nanohydrogels have also been developed from pMAA and PEG (P(MAA-g-EG)) using free radical solution polymerization and dispersion polymerization to polymerize MAA and methoxy-terminated PEG monomethacrylate. The acrylates were then cross-linked with tetra(ethylene glycol) dimethacrylate [85]. The ability for the gels obtained to undergo large swelling changes over small pH ranges is important in allowing these materials to be used in oral delivery of peptides, especially insulin. In acidic environments, such as that of the stomach, insulin is contained within the gel. On the other hand, at higher pHs that mimic that of the small intestine, the insulin is released from the nanogel. Another benefit of these gels is that the PEG chains within the gel stabilize the insulin, allowing it to maintain its proper activity by precluding it from binding to the ionizable MAA chains in the gel.

In another study, PEG hydrogel nanospheres with and without acrylate-modified horseradish peroxidase (HRP), where in the HRP was functionalized with *N*-hydrosuccinamide-PEG-acrylate (PEG-NHS), were prepared through a reverse emulsion photopolymerization [86]. It was found that the enzyme maintained activity after it was encapsulated within the nanogel spheres and that when the spheres were introduced through a phagocytic process within macrophages, the enzyme responded to oxidative stresses and H_2_O_2_ concentrations. The results of the study reveal the potential that these nanogel spheres have in relation to sensing oxidative stress levels and also for drug screening.

**Figure 5:**
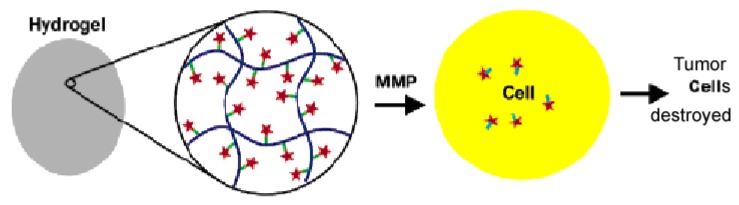
Schematic representation of the proposed local drug delivery system using PEGDA and MMP/ cisplatin. In the figure, cisplatin (red stars) is complexed to peptides (light green lines) pendant on the backbone of a hydrogel matrix (dark blue lines). As matrix metalloproteases diffuse through the matrix, the peptides are cleaved, releasing cisplatin-peptide (aqua lines attached to orange stars) complexes. These complexes may dissociate or enter cells as a complex. Cisplatin is then able to cross-link DNA and have a therapeutic effect only when released from the hydrogel matrix. [87] Copyright, *Reprinted with permission from the American Chemical Society.*

PEG based hydrogels have also been explored for cancer treatment. Hydrogels with mesh sizes at the nanoscale have been developed that can be loaded with cancer therapeutics that may be activated through protease activity, as depicted in Figure 5. 

The hydrogels were synthesized from PEG-DA that also contained peptides sensitive to matrix metalloproteases (MMPs), which are overactive in many tumor cells [87]. Cisplatin, a chemotherapeutic agent, was then either entrapped within the gels or complexed with the MMP-sensitive peptides. The rate of release of cisplatin from the hydrogels was found to be dependent upon the molecular weight of the hydrogel. Higher molecular weight hydrogels released a greater amount of cisplatin due to their larger mesh size. Further, when cisplatin that had previously been encapsulated within the gel was released, it retained its toxicity against the tumor cells, while the complexed cisplatin had lesser activity because it was harder for the drug to be released from the hydrogel. It is also important to note that for higher molecular weight gels, the MMP can diffuse into the hydrogel, where the cisplatin is complexed, and activate the drug. The results obtained from these studies indicate that such hydrogels with nanoscale mesh sizes can be used as injectable drug delivery systems as well as for surgical implantation devices in relation to cancer therapies. PEGDA hydrogels with a nanoscale mesh size have also been utilized in delivering chemotherapeutic drugs to glioblastoma multifore (GBM) tumor cells, which is the most advanced stage of an astrocytic brain tumor [88]. Results indicated that MMPs that are active in GBM would degrade the MMP-sensitive peptides of the hydrogel, allowing the drug to be released at the particular site for which it is targeted, and cisplatin was found to retain activity, even after encapsulation within the gel and further degradation of the peptides incorporated into the gel matrix. The results of the study are of particular importance because current treatments for GBM are not very effective due to the problem of successfully transporting drugs across the blood-brain barrier.

#### 2.2.1. Polyethylene glycol (PEG) Based Hydrogels in Tissue Engineering

PEG hydrogels have also found several applications in tissue engineering. Recently, PEG-DA-based hydrogels were synthesized at their equilibrium water content using a photolithographic method and murine 3T3 fibroblasts were encapsulated within these microstructures [89]. The hydrogels encapsulating murine 3T3 fibroblasts were fabricated specifically using proximity photolithography. In order to do so, PEG-diacrylate (PEG-DA) precursor containing the 1-phenyl-2-hydroxy-2-methyl-1-propanone photoinitiator was mixed with a cell suspension in cell culture media. The fibroblasts were then cultured and incubated until near confluence and transferred back to cell culture media followed by addition to the gel precursor. The cell-containing polymer suspension was spin-coated onto functionalized substrates to form uniform fluid layer. The layer was then covered with a photomask and irradiated with UV light through the photomask. Upon exposure to UV light, only exposed regions underwent free-radical-induced gelation and became insoluble water. The gels were covalently attached to the substrate (with the help of methacrylate moieties and Si-O-Si bonds at the substrate surface). The researchers found that the fibroblasts were encapsulated within the matrix of the cylindrical microgels, rather than on the exterior of the gel, and also that approximately 80% of the cells retained viability within the gel, which is a promising result (similar results were also obtained when the experiment was conducted with murine SV-40 transformed hepatocytes). Overall, the results of this study hold promise in encapsulating live cells within hydrogels, which could prove important in future tissue engineering applications.

In addition, UV-based imprint lithography methods have been developed in order to structure the surface of micro- and nanohydrogels, with the intention that these materials can be used in biomedical research in relation to tissue engineering. The basic material that was utilized is PEG-based star-shape polymers (star PEGs) that contain six arms with a mixture of 4:1 ethylene oxide to propylene oxide that can be end-functionalized [90]. When these arms are functionalized with acrylate (Acr), the Acr-star PEGs can be cross-linked and through the use of the UV-based imprint lithography, nanostructuring can be completed on those gels. Briefly, the lithography technique involved a primary, hard master material, which is used to produce an elastomeric mold, which was then used as a secondary mold to imprint the star PEGs. The bulk star PEG materials hold promise in relation to tissue engineering because they have antifouling properties that would prevent cells from attaching to the surface of an implanted biomedical device. Further research will indicate the type of micro- and nanostructures that would be most advantageous on the surface of these materials.

**Figure 6:**
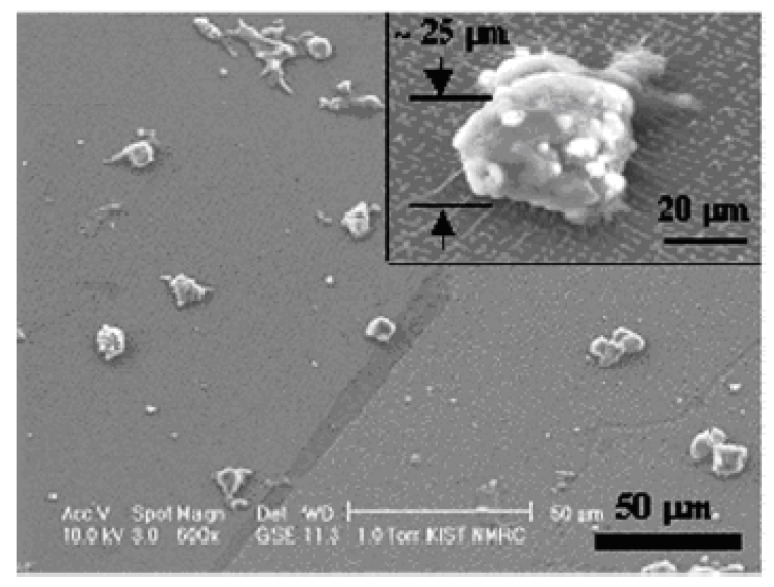
ESEM images of a representative sample of aggregated cardiomyocytes cultured on the PEG nanopillars. [91] Copyright, *Reprinted with permission from the American Chemical Society*.

Researchers have also synthesized PEG cone-shaped nanopillars by using a poly (urethane acrylate) (PUA) mold made with a base of polyurethane cross-linked with acrylate combined with a monomeric modulator and a photoinitiator. The nanopillars that were formed were used in culturing three-dimensional cardiomyocytes, as shown in Figure 6, and it was observed that the nanopillars helped in guiding the extension of the membranes of the cultured cells. The cultured cells displayed significant beating capability, which was tested through electophysical measurements, such as whole-cell patch clamp recording, and which is also very important in relation to the applicability of the cardiomyocytes in cell implants and tissue engineering [91].

Alternating multiblock copolymer gel materials have also been developed that respond to external temperature changes, which could prove important in using these nanoscale gels as injectable biomaterials. The PEG/poly(L-lactic acid) (PEG/PLLA) multiblock copolymer has been synthesized through a coupling reaction between the two polymers. The gelation mechanism for this material was similar to micelle aggregation (with micelles of approximately 20 nm at low temperature forming) as the hydrophilic PEG and the hydrophobic PLLA components formed a core-shell structure with the hydrophilic component forming the shell of the structure facing the aqueous environment. Results showed that there was a reverse thermal gelation between 30-45°C and that the overall molecular weight as well as the relative composition of PEG/PLLA in the gel altered the temperature at which the materials would shift from sol to gel. More specifically, the gels with a molecular weight of 6700 Daltons showed maximal elasticity at physiological temperature. Due to these results, these gels hold promise for uses in injectable biomaterials [92].

### 2.3. Polyphosphazenes Hydrogels in Drug Delivery

**Figure 7:**
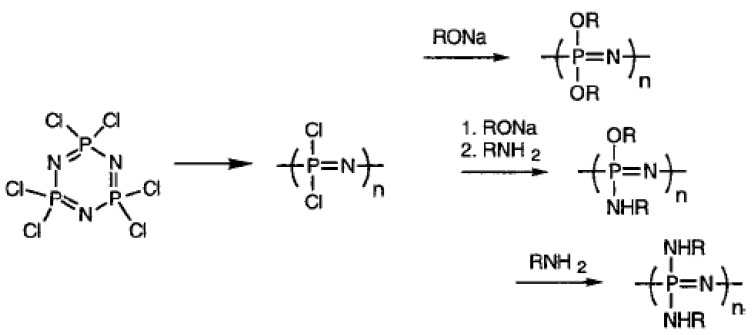
Formation of poly(phosphazenes) and examples of backbone modification. [95], *Copyright*, *Reprinted with permission from The American Chemical Society.*

Phosphorus containing polymers, (polyphosphazenes) have been cross-linked to form microsphere gel structures that can be utilized in drug encapsulation [93,94] (as shown in Figure 7). For example, poly [bis(carboxylatophenoxy)phosphazene] has been cross-linked in the presence of Ca^2+^ ions and the hydrolytic degradation of the gel microspheres based on calcium cross-linked phosphazene polyelectrolytes, poly[di(carboxylatophenoxy)phosphazene] (PCPP) and poly[(carboxylatophenoxy) (glycinato)phosphazene] (PCGPP) have been studied [95]. Both PCPP and PCGPP ionotropic polyphosphazene hydrogels were found to be degradable in aqueous environments and the degradation rates could be increased by incorporation of hydrolysis sensitive glycinato groups into the polymer (PCGPP). Such ionotropic polyphosphazene hydrogels can be utilized as potential biodegradable devices for controlled drug delivery systems. Polyphosphazene–hydroxyapatite (HAp) composites have been synthesized via acid–base reactions of tetracalcium phosphate and anhydrous dicalcium phosphate in the presence of polyphosphazenes bearing alkyl ester containing side-chains such as poly(ethyl oxybenzoate)phosphazene (PN-EOB) and poly(propyl oxybenzoate) phosphazene (PN-POB). The composite materials formed were found to be biodegradable and may be suitable as bone analogs [96]. Poly(organo phosphazene) nanoparticles surface modified by adsorption of poly(organo phosphazene)-poly(ethylene oxide) copolymer with a 5,000 M_w_ PEO chain (PF-PEO_5,000_), have also been synthesized and found to be highly biodegradable. Studies of the biodistribution of the PF-PEO_5,000_-coated poly(organo phosphazene) nanoparticles in the rabbit model also indicated a prolonged systemic circulation lifetime and reduced liver uptake [97].

### 2.4. Acrylic Acid Hydrogels for Drug Delivery in Treatment of Diabetes

Another group of materials utilized in possible drug delivery applications in relation to diabetes are amphoteric microgels composed of acrylic acids (AA) (an anionic monomer) and *N,N*-dimethyl amino ethyl acrylate (a cationic monomer) that have been functionalized with phenylboronic acid [98]. The two monomers chosen to synthesize the gel tend to co-localize within the gel network because of their kinetic properties [99,100], which is important in allowing their amino groups to interact with the boronic functional groups. These materials have been synthesized to respond to increases in glucose concentration and pH by either shrinking or swelling. Futher, at a threshold glucose concentration, the surface charge of the gels can be converted from cationic to anionic, causing insulin bound within the gel to be released. This system allows a glucose-sensitive insulin release system to be designed for utilization within the body. 

### 2.5. Poly(methacrylic acid) (PMAA ) in Cancer Treatment and Drug Delivery

Methacrylate derivatized poly(vinyl-alcohol) (PVA-MA) has been cross-linked in the presence of dextran and further subjected to an emulsion process in aqueous solutions based on polymer-polymer immiscibility in order to form micro hydrogels. These microparticles were then coupled with succinic anhydride to give them a larger negative charge. The microhydrogels were loaded with doxorubicin (DOX) and targeted at cancerous human colon cells. A decrease in the number of cancerous cells was observed, which indicates that these structures hold promise in relation to future drug delivery systems for cancer treatment [101]. Anionic microgels, synthesized from methylene-bis-acrylamide and MAA in a 1:4 mole ratio, were loaded with DOX and coated with a lipid membrane composed of dipalmitoylphosphatidylcholine, dipalmitoylphosphatidylglycerol sodium salt, and cholesterol (4:1:5 mole ratio) [102]. The membranes were added to the microgels in order to prevent the microgels from swelling and releasing the DOX in physiological media unless the membranes become porated, which is a mimic of the membrane of secretory granules fusing with the plasma membrane of the target cell. It was found that the DOX would only be released by breaking down the outer lipid membrane, a process labeled “triggered burst release,” which has potential applications if the gels are reduced in size to nanohydrogels in the release of drugs into tumor tissue. 

**Figure 8:**
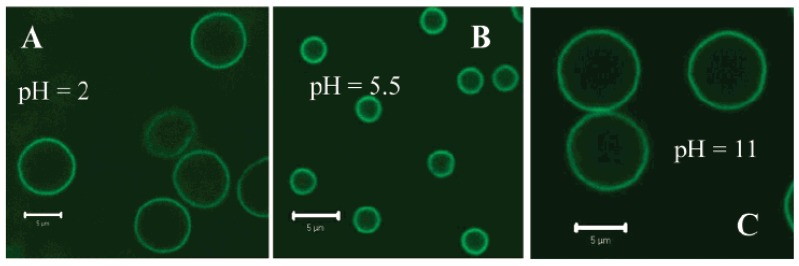
pH dependence of the diameter of the (PMAA)_7_ capsules cross-linked for 22 h. CLSM images show the (PMAA)_7_ capusles at pH = 2 (A), pH = 5.5 (B), an pH = 11 (C). The scale bar is 5 μm [103] *Copyright, Reprinted with permission from the American Chemical Society.*

PMAA has also been coupled with poly(*N*-vinylpyrrolidone) (PVPON) [103] and the hydrogen-bonded multi-layers of this material were deposited in cycles onto silica microparticles. The deposited layers were cross-linked with ethylenediamine, and then the microparticle core was dissolved with an aqueous HF solution, which led to the formation of a hollow capsule composed of the (PVPON/PMAA) multilayer material. When the level of cross-linking was altered, the pore size of the microgel was also altered, which consequently changed the types of materials that could be taken up by the gel. Results revealed that the amphoteric capsules produced were sensitive to both pH and ion concentration of the external environment and depending on the surface that the capsule came in contact with, the soft capsule could flatten itself, as shown in Figure 8. The capsules remained spherically shaped unless they were in direct contact with a solid surface such as a glass coverslip. Upon contact with a solid surface, they could either flatten, acquiring a pancake like shape, or remain spherical. It was also seen that highly cross-linked (PMAA) capsules retained their spherical shape under mildly acidic conditions but were completely flattened under highly acidic conditions when in contact with bare glass. No flattening was observed for capsules, which were not in direct contact with a surface, for example, those, which precipitated on top of the first “layer” of surface-adhered capsules. This suggests that the flattening is mainly induced by interaction of the capsule walls with the surface and is a combination of electrostatic interactions between positive charges and pH dependence of the diameter of the (PMAA)_7_ capsules cross-linked for different periods of time. Based on the responses to pH, it was found that at acidic and basic pH, macromolecules could be loaded into the capsule, while at around pH 5.5, the capsule would no longer uptake material. In order to release the materials, the capsule needed to be placed into a high salt environment. These results reflect the ability of these microgel capsules to be utilized in the selective uptake and release of drugs and other macromolecules as a useful container in delivery systems.

### 2.6. Polyvinylamine Hydrogels in Drug Delivery

**Figure 9:**
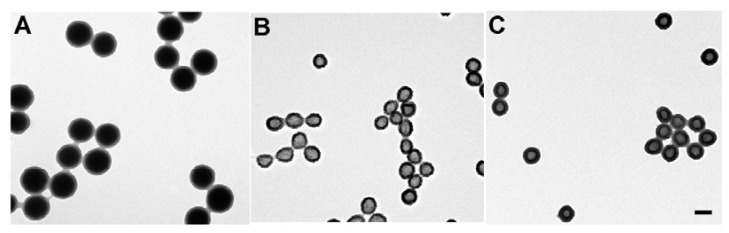
TEM images of the PVAm capsules. (A) PVNF shell/silica core composite particles: PNVF shell thickness: 18 ± 1.0 nm; core diameter: 127 ± 6.2 nm. (B) PNVF capsules: PNVF shell thickness: 19 ± 1.0 nm; core diameter 68 ± 7.3 nm. (C) PVAm capsules: PVAm shell thickness: 23 ± 0.5 nm; core diameter 57 ± 3.9 nm. Scale bar = 100 nm. [104] Copyright, *Reprinted with permission from the American Chemical Society*.

Another material that is being investigated in relation to as yet non-specified drug delivery systems is polyvinylamine (PVAm). The acid-labile PVAm nanogel capsules that have potential in drug delivery are synthesized through hydrolysis of a poly (*N*-vinylformamide) (PNVF) shell that was produced from etching shell/silica core nanoparticles of PNVF, as shown in Figure 9. It was found that as the pH of the environment surrounding the PVAm nanogel capsules was decreased, the swelling of the material increased mainly due to an increased number and charge of the protonated amine groups of the capsule. This allowed for the cross-linking of the polymer network to stretch and expand, leading to increased swelling. This result proves important in the potential to load these capsules with drugs or other therapeutics [104].

### 2.7. Poly (adipic anhydride) Microgels in Drug Delivery

Poly(adipic anhydride) is an aliphatic anhydride, which has been used in combination with several other polymers for the preparation of micro and nanogels for controlled drug release. Recently, poly trimethylene carbonate (PTMC), and poly(adipic anhydride) were synthesized via melt condensation and ring-opening polymerization of trimethylene carbonate and adipic acid, respectively [105]. The release of clomipramine HCl and buprenorphine HCl from discs prepared with the use of PTMC-PAA blends in buffer solutions was investigated. It was found that for devices containing 50% and more poly (adipic anhydride), surface erosion played a significant role in the release of the drugs studied. Poly (adipic anhydride) has also been encapsulated with an ocular drug, timolol maleate, and investigated for suitable use as a drug delivery system. The goal was to increase the precorneal residence time of ocular drugs and it was expected that by combining the gel with a microparticle, water-soluble drugs will not be released as quickly as they would by just encapsulating them within a hydrogel. By studying the degradation products formed when the microsphere-gels degraded, it was determined that adipic acid was released, indicating that the surface of the particle was being degraded. While the use of this system in drug delivery to the eye had yet to be compared to ordinary eye drops, there is promise that it will be suitable in biocompatible ocular drug delivery [52].

### 2.8. Poly(N-vinyl formamide)in Enzyme Encapsulation and Drug Delivery

Another material that has been studied is the water-soluble and acid-labile polymer poly(*N-*vinyl formamide), which has been cross-linked to form hydrogel nanoparticles through an inverse micro emulsion polymerization [106]. It was discovered that using these materials, the polymerization of the nanogels could occur at low temperatures (35 °C), which is important in allowing proteins to be encapsulated within the gels without the occurrence of misfolding. The nanogels were found to be minimally cytotoxic when the enzyme lysozyme was encapsulated within this material, and the enzyme further retained an average of half of its activity compared to the activity of the enzyme outside of the nanogel. The results of the study show promise for the use of these nanohydrogels intravenously in biocompatible protein delivery.

### 2.9. Pluronics in Drug Delivery

**Figure 10:**
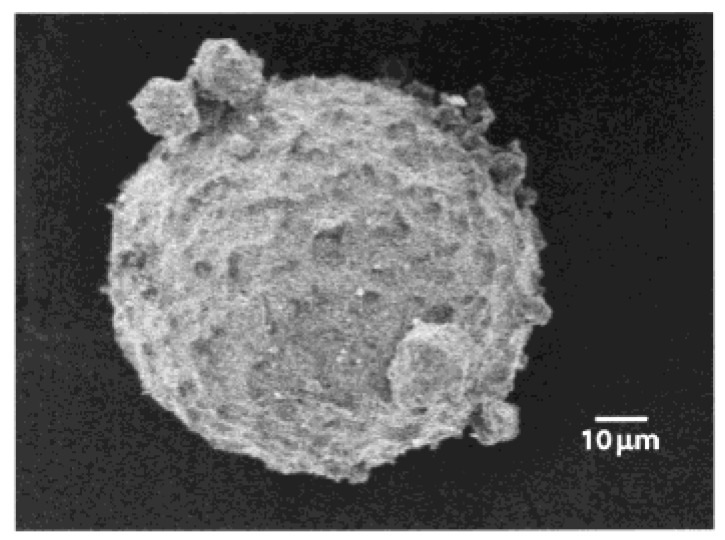
SEM image of the Pluronic-PAA-EGDMA particles with effective cross-linking degree. The particles shown were removed from the reactor, washed with hexane, and dried. [108] Copyright. *Reprinted with permission from the American Chemical Society.*

Some of the most valuable block copolymers are the amphiphilic block copolymers poly(ethylene oxide) − poly(propylene oxide) − poly(ethylene oxide), PEO*_y_*PPO*_x_*PEO*_y_* (Poloxamers (ICI) or Pluronics (BASF)). Pluronics are known to self-assemble in water into micelles consisting of a hydrophobic core of PPO surrounded by solvated PEO [107]. Recently, microparticle gels consisting of cross-linked poly (acrylic acid) with ethylene glycol dimethacrylate (EGDMA) were synthesized and poly(ethylene oxide)-poly(propylene oxide)-poly(ethylene oxide) (PEO-PPO-PEO, Pluronics) copolymers were grafted onto the micro particles [108]. An example of the types of particles obtained is shown in Figure 10. Results indicated that at higher temperatures, the pluronic chains rearranged forming aggregate structures that assisted in cross-linking the gel, which lowered the equilibrium swelling of the microgel. These aggregates were able to dissolve hydrophobic drugs, and the carboxyl groups of the microgel particles could also help with the uptake of cationic drugs into the gel. This is important because both taxol and DOX, anticancer agents, can be loaded into these microparticles, which can then be used in oral or topical drug delivery applications.

**Figure 11:**
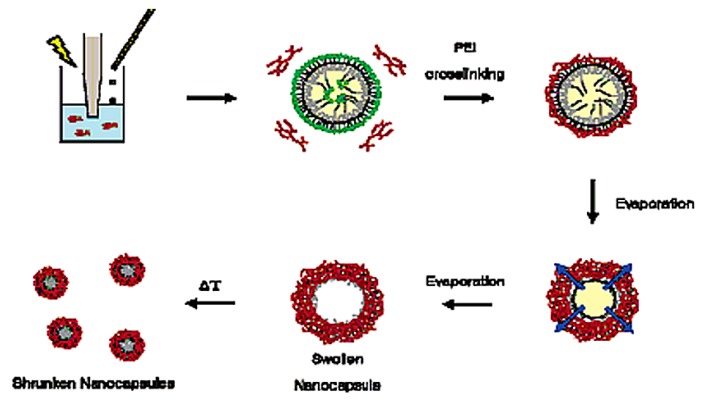
The steps in the emulsification and solvent evaporation combination method used to prepare Pluronic/PEI nanocapsules. [109] Copyright *Reprinted with permission from the American Chemical Society.*

Hydrogels synthesized from the amphiphilic triblock copolymer, pluronic, which was pre-activated with an amine-specific reactive group (Pluronic F-127), and poly(ethylenimine) (PEI) through an emulsification and solvent evaporation combination method, are shown in Figure 11. It was shown that those hydrogels underwent a deswelling process as temperature was increased (at 20 °C the nanocapsules were 330 nm in size, while at 37°C they were 100 nm), and that the nanogel capsules were also hollow, allowing for them to be loaded with various materials [109]. The nanocapsules can be taken up by cells through endocytosis and retained within an endosome until a cold-shock is applied to the cell, upon which the nanocapsules broke out of the endocytic compartment. Such biocompatible hydrogel nanocapsules can be potentially utilized in the delivery of cancer therapeutics and other anti-cancer agents directly to target cells. 

## 3. Supramolecular Hydrogels in Drug Delivery

**Figure 12:**
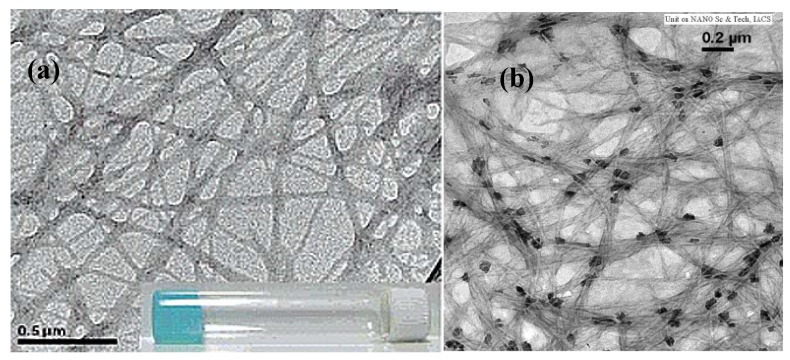
(a) TEM image of the metallo-hydrogel prepared from bolaamphiphile (sebacic/azelaic acid coupled with methyl esters of l-Phe)-Cu salt at pH 6.5; (b) TEM image of the metallo-hydrogel prepared from bolaamphiphile-Co salt at pH 6.5 in the presence of vitamin B_12_ showing the trapped vitamin molecules within the gel nanofiber network. [112] Copyright, *Reprinted with permission from the American Chemical Society.*

Supramolecular hydrogels developed from self-assembling nanofibers made from bioactive materials, such as vancomycin and glucosamine, hold promise because the bioactivity of the molecules remains unaffected or is even enhanced when altered to the hydrogel state. In some cases, the vancomycin was conjugated with pyrene due to pyrene’s ability to form dimers or oligomers in solution as well as render the hydrogels fluorescent [110]. It was found that those self-assembling nanofiber gels formed had hydrophilicity, which was a balance between the hydrophobic (from pyrene) and hydrophilic (from vancomycin) domains of the molecule. In another study, shell-crosslinked nanospheres have been synthesized based on shell-crosslinked knedel-like structures (SCKs) with PEO stabilizing the outer layer of the spheres. The PEO links in the outer layer give the spheres a thicker hydrogel-like activity in that layer, allowing the spheres to be utilized in drug delivery systems [111]. For potential use in the uptake and release of vitamins within the body bolaamphiphile-based smart metallo-hydrogels with a nanofiber network have also been developed as shown by Banerjee and co-workers [112]. They synthesized bolaamphiphiles by connecting methyl ester functional groups of amino acids with sebacic/ azelaic acid, and in the presence of metal salts, such as CuSO_4_, CoCl_2_, MnCl_2_, and NiCl_2_. The bolaamphiphiles were found to self-assemble into hydrogels. Results indicated that the various nanofiber metallo-hydrogels that were formed were pH responsive, with their highest stability at physiological pH (6.5 – 7.2). Further, the solubilities of the metallo-hydrogels in water were also found to be highly pH sensitive. It was observed that if the hydrogels were formed in the presence of vitamin B_12_, the vitamin became encapsulated within the nanofiber network. However, when the pH of the environment was changed, the release of the vitamin B_12_ could be controlled. The structures of the self-assembled nanofiber hydrogels are shown in Figure 12. Thus, these metallo-hydrogels promise a novel way of constructing hydrogels and of delivering molecules, such as various vitamins, within the body.

## 4. Carbohydrate Hydrogels in Biomedicine

**Figure 13:**
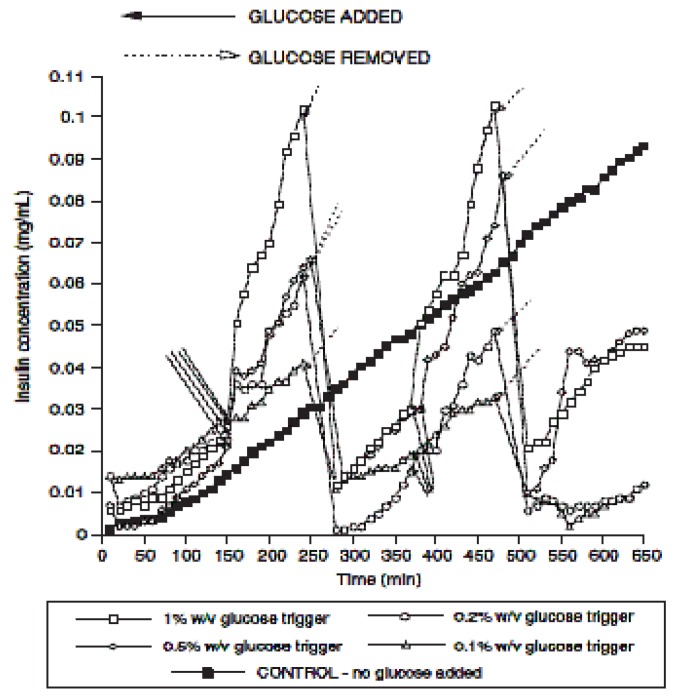
Insulin release profiles in glucose dose response studies for a 70 min irradiated mixture of dex-MA (DS 3%)-con A-MA in a double glucose trigger experiment. [114] Copyright, *Reprinted with permission from Elsevier.*

Microgels consisting of dextran derivatives cross-linked with the lectin concanavalin A (ConA) have been incorporated with insulin [113,114]. It was observed that the insulin was released in higher quantities when there was a high concentration of glucose present, because the dextran and glucose compete for binding sites with ConA, which tears the gel and releases the insulin. The release profile for insulin is shown in figure 13. Other natural materials such as chitosan and ovalbumin have also been used to synthesize nanogels that can be utilized in drug delivery systems [115]. It is well known that chitosan and its derivatives are elastomeric, biocompatible, antibacterial and resorbable [116,117,118,119]. The benefits of such nanogel spheres include having the biomedical assets of chitosan as well as the ability to synthesize these particles in a green way that does not require separation during preparation. 

The nanogels were prepared by titrating chitosan in acetic acid solution (0.75% chitosan in 0.75% acetic acid, w/v) into an ovalbumin aqueous solution, while agitating the mixture. After stirring, adjusting the pH, and heating the solution, nanogels were formed with chitosan chains partly held within the core of the ovalbumin gel and the rest of the chains forming a shell around these nanogel spheres. It was found that the nanospheres had a pH-dependent hydrophobic/hydrophilic property. At basic pH the nanospheres could uptake cationic drugs, while at an acidic pH the drugs would be released because the gel itself will also hold a positive charge. This is important in using these nanoparticles in developing potential drug delivery systems based on electrostatic attractions.

Chitosan has also been utilized in forming nanoscale hydrogel-based materials that can be used in biosensing applications [120,121]. In a recent study, gold nanoparticles were diffused within a chitosan solution (0.5%) that also contained glucose oxidase and then an electrodeposition procedure was utilized in order to from a “hydrogel-based nanoparticle-enzyme biocomposite.” The chitosan lends its biocompatible aspects and its ability to be deposited as a hydrogel to the material and the gold nanoparticles were found to stabilize the material and detect the H_2_O_2_ that may be liberated by glucose oxidase activity [122]. It is important to note that enzyme activity was retained upon being incorporated into the material and the results also indicated that the biocomposites may be useful as biosensors because they could detect certain reaction products. Recently, a solution of modified chitosan, *N*-[(2-hydroxy-3-trimethylammonium)propyl]chitosan chloride (HTCC), was combined with sodium tripolyphosphate (TPP) in order to prepare the microgels and methotrexate disodium (MTX), a cytotoxic drug utilized in the treatment of cancer, was loaded into the gel [123]. Further, the gels were conjugated with apo-transferrin, because this protein is able to enter cells through receptor-mediated endocytosis. During this process, the hydrogels, undergo a lowering of their pH from 7.4 to 5.0 within the cell, which enhances the speed at which the MTX would be released into the lower pH environment in the tumor cells. At pH 5.0, the MTX will most likely not denature as previous studies have shown that MTX is hydrolyzed only in strongly acidic or strongly alkaline conditions to give glutamic acid and 4-amino-4-deoxy-10-methylpteroic acid [124]. 

Pullulan, a polysaccharide consisting of maltotriose units has been combined with cholesterol to form self-aggregating hydrogels. The cholesterol groups allow cross-linking within the gel to occur. Also, cholesteryl-group bearing pullulan (CHP) was shown to bind to hydrophobic substances and proteins. Studies have shown that when proteins such as chymotrypsin, BSA or insulin were complexed to the CHP hydrogels, even though the higher order structure of the protein was altered, the conformation of the complexed protein did not change significantly when the nanoparticles were subject to various denaturing conditions, including 9 M urea and high temperatures (92ºC). Self-aggregation of the BSA was also prevented with complexation to CHP [125,126,127,128]. These findings display promise for the use of CHP self-aggregating hydrogel nanoparticles in medicine because the hydrophobic polysaccharide, CHP, is able to complex with other molecules, helping to stabilize them against changes in the external environment. Pullulan derived hydrogel nanoparticles have been found to be important in preventing the denaturation of enzymes, and aiding in renaturing them. In a recent study, carbonic anhydrase B (CAB) that was denatured from heat exposure, revealing its hydrophobic segments, was complexed within the CHP nanohydrogels [129]. After adding β-cyclodextrin to the CHP nanohydrogels complexed with CAB, the cross-links in the CHP particle were broken due to the presence of the β-cyclodextrin and CAB was released from the CHP nanohydrogel in refolded form. These results indicate that thermal stability of certain enzymes can be drastically increased by complexing them with CHP self-aggregating nanohydrogels, which act as molecular chaperones, because CAB usually undergoes an irreversible denaturation when exposed to heat. This is promising and CHP hydrogel nanoparticles may find future use in protein and enzyme delivery.

One benefit of such materials is that the network within the gel prevents aggregation of the protein molecules, which stabilizes them for delivery within the body. Cationic nanogels composed of cholesteryl group-bearing pullulans functionalized with an ethylenediamine group (CHPNH_2_), which are amphiphilic polysaccharides, are one such material being synthesized for utilization in protein delivery [130]. Proteins such as bovine serum albumin and β-galactosidase were encapsulated within the nanogels and it was found that once the nanogel particle was taken up by the cell, the nanogel-protein complex dissociated and the protein was released. Self-organizing nanogels have also been studied in relation to anti-tumor drug delivery systems. Pullulan-deoxycholic acid (PUL-DO) conjugates have also been synthesized from dicyclohexylcarbodiimide (DCC)- and 4-dimethylamino-pyridine (DMAP)-mediated ester formation, and histidine was further conjugated onto the formed PUL-DO conjugates [131]. After self-assembly of the PUL-DO/His conjugates into nanogels through a dialysis method, the anticancer drug DOX was loaded into the gels. It was found that at pH 6.2, the size of the nanogels drastically increased compared to the size at pH 8.5 because the His imidazole ring was ionized, causing destabilization within the gel. This is important because tumor pH is around 6.8, and at this pH, a higher cytotoxicity because of the enhanced drug release rate was observed due to the increase in gel size. These experimental studies need to be considered if such nanogels are to be used as anti-tumor agents, especially in relation to breast cancer cell lines.

Carbohydrates and their derivatives such as glycosaminoglycans can also be utilized to synthesize microscale hydrogels that hold promise in relation to tissue engineering. In order to synthesize the hydrogels, various concentrations of carboxymethylcellulose sodium salt (CMCNa), (in order to give pH and ion sensitivity to the gel), hydroxyethylcellulose (HEC), (to increase intermolecular cross-linking within the gel), and hyaluronic acid were cross-linked with divinyl sulfone (DVS) under basic conditions [132]. Results indicated that when there was a decrease in the cross-linking of the gel or an increase in the ionic group concentration (due to higher CMCNa concentration), there was an increase in the amount of water that the gel was able to absorb. All of the gels, regardless of the concentration of hyaluronic acid or cellulose, responded to changes in external ion concentration. The results obtained from these studies reveal that such natural material microscale gels can be utilized as “barrier substances” to prevent tissue adhesion after surgical procedures.

**Figure 14:**
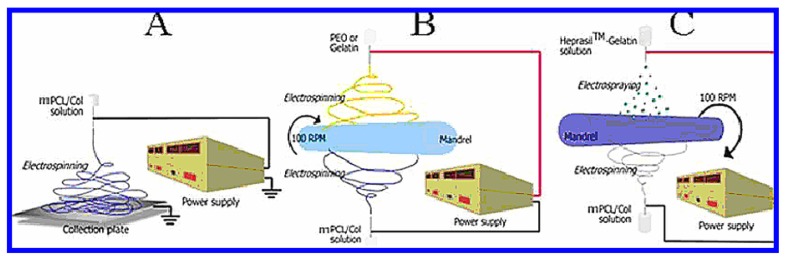
Three separate electrospinning setups utilized in forming hydrogels: conventional flat plate collection (A), two-capillary coelectrospinning system (B), and two-capillary electrospinning-electrospraying system (C). In all three systems, mPCL/Col was used as the main fiber material. PEO or gelatin was used as water-soluble fiber material for selective leaching approach (B). Heprasil hydrogel was embedded in the mPCL/Col mesh using a simultaneous electrospraying-electrospinning setup (C). [133] Copyright, *Reprinted with permission from the American Chemical Society*.

Electrospinning has also been used to design hydrogel scaffolds with applicability in tissue engineering, but there have been problems with integrating the electrospun scaffold into the extracellular matrix. In order to attempt to overcome these difficulties, micron-sized fibers of poly(ε-caprolactone)/collagen (mPCL/Col) were used in the electrospinning in order to increase the distance between fibers and the size of the pores formed within the hydrogel. A second method utilized to overcome the integration problems was combining the electrospun μmPCL/Col fibers with electrosprayed Heprasil, which is modified hyaluronic acid, a glycosaminoglycan component of the extracellular matrix [133], as shown in Figure 14. It was found that both of these materials were biocompatible and increased the integration of the hydrogel scaffold within the cell structure. The Heprasil-μmPCL/Col hydrogel presented an added benefit to its use as a tissue scaffold in that it could also be used in the release of drugs or other biocompatible materials.

## 5. Peptide Hydrogels 

**Figure 15:**
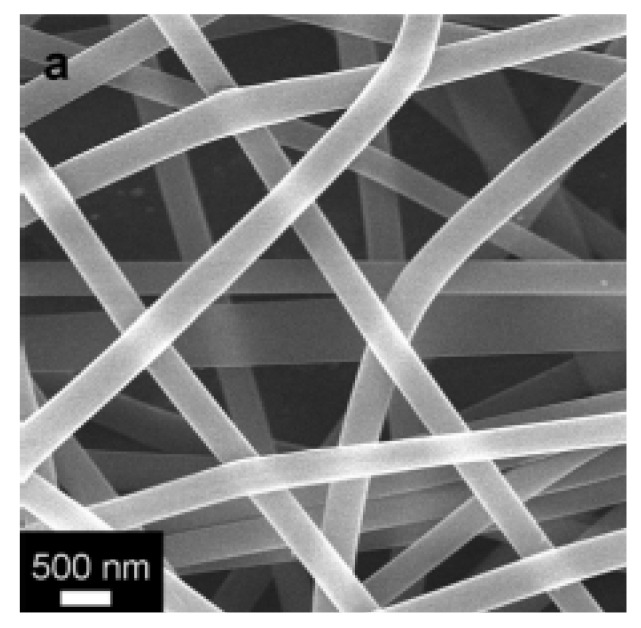
SEM image showing the PVA/ferritin nanofiber hydrogel in the dry state. [135] Copyright *Reprinted with permission from the American Chemical Society.*

Novel techniques in the engineering of neural tissue have also stemmed from the use of nanoscale hydrogel materials. Researchers have found that peptide hydrogels with a fibrous structure at the nanoscale have been formed through self-assembly processes when the peptide components such as arginine, alanine, and aspartate, are left under physiological conditions. These self-assembled peptide nanofiber scaffolds (SAPNS) are especially useful in tissue engineering because the nanofiber network mimics the extracellular matrix and can incorporate itself with minimal or no immunological response into the matrix of cells within the body. It has been found that functional synapses in rat hippocampal neural tissue have formed in the presence of these scaffolds, which leads to promising applications of these nano-hydrogels in further neural tissue development [134]. Another study, in relation to nanogel applications in tissue engineering involved the synthesis of poly(vinyl alcohol) (PVA)/ ferritin (iron containing protein) nanofibers using an electrospinning technique, and then placing the nanofibers on an aluminum electrode (cathode) in methanol to allow the fibers to cross-link and form a gel. The structures of the formed fibers are shown in Figure 15. Such ferritin complexes are not only biocompatible, because ferritin complexes are found in organs such as the liver, spleen and the brain, but also give the gel super paramagnetic properties, which is beneficial in constructing cartilage and cell scaffolding implants that are easier to visualize through MRI scans [135].

**Figure 16:**
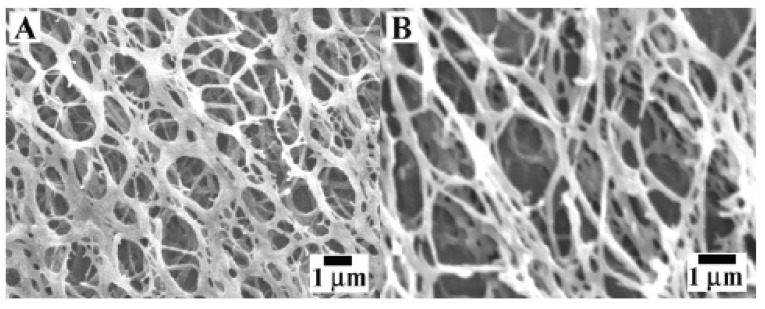
SEM images of hydrogel formed using varying concentrations NPC 15199, and Fmoc-L-lysine, in the absence (A) and presence (B) of 5-fluoro-2’-deoxyuridine. [52] Copyright (*Reprinted with permission from The Royal Society of Chemistry*).

Composites of gelatin (a protein formed by the partial hydrolysis of collagen) have also been used as hydrogels and have found biomedical applications. For example, gelatin-silica hybrid capsules have been developed and have been found to display high cyto-compatibility. It was seen that as the biopolymer component of the capsule was taken in and degraded by the cell, the hollow silica particles remained intact. These gelatin-silica capsules were prepared using a technique similar to that used to synthesize microscale alginate-silica capsules, where in first the alginate particles were coated with poly(L-lysine) (PLL) to make the surface of the particle positively charged. In the second step, silica was deposited. In the case of the gelatin-silica capsules, the gelatin took the place of the alginate core during the synthesis. The microscale alginate-silica particles were comparatively difficult to synthesize, and when gelatin was utilized in the sol-gel process, it was easier to change the porosity of the silica and form micro- and nanoscale capsules, which have potential applicability in future drug loading and delivery systems [139]. Recently Xu and co-workers synthesized nanoscale hydrogels from *N*-(fluorenylmethoxycarbonyl) amino acids, such as NPC 15,199 and Fmoc-l-lysine. It is believed that those hydrogels could potentially possess anti-inflammatory activity [52] and can act as carriers for bioactive agents by simply adding the agents into the precursor amino acids. They observed that upon the addition of 5-fluoro-2’-deoxyuridine the hydrogel fibers formed were thicker, indicating the incorporation of 5-fluoro-2’-deoxyuridine within the hydrogels as shown in figure 16. 

Nano- and microscale hydrogel materials are also being studied due to their potential use in designing artificial muscle. The hydrogel material was combined with silicon nanocolumns (known as high-aspect-ratio rigid structures [AIRS]) because the AIRS provide structure and stability to the material, while the hydrogel aspect allows the material to be responsive. The hydrogel component utilized in producing these materials was synthesized from polyglycidyl methacrylate (PGMA) modified with AA and further combined with a pAm gel layer. By embedding the nanocolumns into the hydrogel, in either a free-standing or attached fashion, and activating them into a controllable microstructure, it was found that the actuation of the material was fast, reversible, and reproducible, which holds promise in relation to developing artificial muscle material, as well as release systems [137].

## 6. Dendrimers in Drug Delivery

Linear macromolecules occasionally contain some small or longer branches. Over the years, it has been found that the properties of highly branched macromolecules are different from conventional polymers. The structure of these materials also had a great impact on their applications. First discovered in the early 1980’s by Tomalia and co-workers, such hyperbranched molecules were called dendrimers [138]. Dendrimers, hold potential in being utilized at the nanoscale in hydrogel drug delivery systems. Dendrimers that are functionalized with Girard-P and Girard-T reagents as end groups can form nano-hydrogel networks when dissolved in water at low concentrations and then either heated for a few hours or left at room temperature for a few days. Interaction among the end groups of these phosphorous dendrimers form gels that are very rigid, but can hold large pockets of water. Due to the fact that when these gels are formed, they have the ability to encapsulate various substances, these materials have the potential to be used in the targeted delivery of certain materials, such as drugs or other active molecules [139]. Other attempts to use dendrimers in the targeted delivery of drugs and other therapeutics have also been carried out. The drug molecules can be loaded both in the interior of the dendrimers as well as attached to the surface groups. For example, sialylated dendrimers, have been shown to have potent inhibitory activity toward haemagglutination of human erythrocytes caused due to the influenza virus. It has been shown that the sialytaed dendrimers can attach through the interactions with the receptor of the virus [140]. The sialodendrimers bind to haemagglutinin and thus prevent the attachment of the virus to normal cells. Therefore, they can be useful therapeutic agents in the prevention of bacterial and viral infections. As the degree of silation was enhanced, the potency was found to increase [141]. Water-soluble dendrimers have been found to efficiently bind to solubilizing small hydrophobic molecules with antifungal or antibacterial properties and bound substrates are released upon contact with the target organism [142,143]. Dendrimers have also been used as coating agents to protect or deliver drugs to specific sites in the body or as time-release vehicles for biologically active agents. For example, 5-Fluorouracil (5FU) is known to have significant antitumour activity, but it has high toxic side effects. Zhou and co-workers synthesized acetylated PAMAM dendrimers forming dendrimer-5FU conjugates [30]. The dendrimers were found to be water-soluble and hydrolysis of the conjugates released free 5-fluorouracil units. The slow release of the drug reduces its toxicity. Such dendrimers may have potential applications as antitumor drug carriers [144]. Recently, first generation, photocrosslinkable dendrimers consisting of natural metabolites such as succinic acid, glycerol, β-alanine and poly(ethylene glycol) (PEG) were synthesized using ester and carbamate forming reactions [145]. In the next step, aqueous solutions of those dendrimers were photocrosslinked with an eosin-based photoinitiator to form hydrogels. The hydrogels formed showed minimal swelling characteristics. The dendrimer solutions were then photocrosslinked in situ in an *ex vivo* rabbit osteochondral defect, and the resulting hydrogels were subjected to physiologically relevant dynamic loads. Magnetic resonance imaging (MRI) showed the hydrogels were fixated in the defect site after the repetitive loading regimen. Further, the hydrogel-treated osteochondral defects showed good attachment in the defect site. Thus such dendrimer-based hydrogels can be utilized as scaffolds for osteochondral defect repair.

## 7. Toxicity Studies of Hydrogels

The possible toxicity of hydrogels is an important issue to be considered particularly with respect to biomedical applications. Recently, Haraguchi and co-workers examined clay-hydrogel nanocomposites for the biocompatibility and possible toxicity [146]. They carried out cell cultivation on the surface of PNIPAm/ Clay cross-linked networks with three different cell-lines, namely HepG2 human hepatoma cells, human dermal fibroblasts and human umbilical vein endothelial cells. Their results indicated that the materials were highly biocompatible with those cell-lines and no toxicity was observed. In another study, elastin-mimetic hybrid polymers (EMHPs) were synthesized from PEG with azide-modified end groups and a short peptide consisting of Fmoc-l-alanine, Fmoc-l-lysine, or Fmoc-l-propargylglycine [147]. The EMHPs were covalently cross-linked with hexamethylene diisocyanate (HMDI) in order to form an elastomeric gel that was able to absorb a significant amount of water and was also highly compressible. Toxicity studies were conducted with EMHPs as well as on the cross-linked EMHPs (xEMHPs) in porcine vocal fold fibroblasts (PVFFs). It was found that while the EMHPs alone were slightly toxic toward the fibroblast cells, the cells that came in contact with the xEMHPs were still viable and were able to grow and proliferate after three days of being in culture. Such materials hold promise in relation to using the cross-linked EMHP gels in tissue engineering applications because they would not release any toxic materials into the surrounding cells.

The biocompatibility of poly (2-hydroxyethyl methacrylate) (pHEMA) hydrogels synthesized using atom transfer radical polymerization and the degradable cross-linker, polycaprolactone was also investigated [148]. The hydrogels were placed on mouse fibroblasts and the degradation products of the hydrogel were not found to be cytotoxic toward the fibroblasts. The cytotoxicity of the hydrogels and their degradation products was tested using the MTT (3(4,5-dimethylthiazol-2-yl)-2,5-diphenyl-tetrazolium bromide) assay. Further, the degradation products were found to be soluble in a solution of a comparable ionic strength to blood, indicating that the materials were able to solubilize and be excreted from the body. Qian and co-workers have prepared a biodegradable and pH-sensitive hydrogel based on poly (ε-caprolactone), methacrylic acid, and PEG (p(CL-MAA-EG)) [149]. Due to the combined benefits of biodegradability and pH-sensitivity, these hydrogels are touted to have enormous applications in drug delivery, particularly in oral protein delivery. To ensure that the materials were biocompatible, they carried out *in vivo* studies by acute oral toxicity tests and histopathological observation of BALB/c mice. It was observed that the oral administration of p(CL-MAA-EG) hydrogels up to 15 g/kg body weight had no toxic effects. Further, no significant histopathological changes were observed due to the presence of p(CL-MAA-EG) hydrogels, though p(CL-MAAEG) was found in the GI tract. Thus, such p(CL-MAA-EG) hydrogels are likely to be safe and may potentially be used as oral drug carriers.

Progress in recombinant protein technology has lead to new protein and peptide therapeutics for treatment of diseases [150]. Nevertheless, their effective delivery is exigent due to their high molecular weights and unique three-dimensional structures. In addition, they are prone to proteolytic degradation and consequently extremely short plasma circulation times and rapid renal clearance. Polymeric controlled release formulations like PLGA offer a sustained release mechanism where in the drug release rates can be controlled by changing the polymer molecular weight and composition. However, it is well-known that hydrophobic polymers induce harmful effects to the encapsulated proteins or peptides during network preparation and delivery [151] and sometimes may activate the host immune response [152]. In contrast, hydrophilic hydrogels, provide a relatively mild network fabrication and drug encapsulation conditions that make them appropriate for protein delivery [153]. Hydrophilic hydrogel systems are also useful for soft tissue engineering applications that require a flexible material mimicking the extracellular matrix (ECM) [154]. Such hydrated hydrophilic polymer networks often contain pores and void regions between the polymer chains, which can be conducive to improved supply of nutrients and oxygen for the cells. The pores within the network provide space for cells, and after proliferation and growth, for the newly regenerated tissue [155]. Ferruti and co-workers have prepared biodegradable, biocompatible amphoteric poly(amido-amine) (PAA)-based hydrogels containing carboxyl groups along with amino groups in their repeating unit, as scaffolds for tissue engineering applications [156]. They also synthesized hybrid PAA/albumin hydrogels. The PAA hydrogels were found to be soft and swellable. Cytotoxicity tests with fibroblasts revealed that the amphoteric PAA hydrogels were cyto-biocompatible both as free bases and salts. Degradation tests under controlled conditions replicating biological environments indicated that most PAA samples (except PAA/albumin hydrogels) degraded completely and dissolved within ten days. Further, the degradation products of all samples were non-cytotoxic. The studies indicate that PAA-based hydrogels have potential as degradable matrices for biomedical applications. Schmidt and co-workers synthesized biomimetic hydrogels in order to promote tissue repair using hyaluronic acid (HA) as starting material [157]. They prepared a variety of glycidyl methacrylate-HA (GMHA) conjugates, which were then photopolymerized to form cross-linked GMHA hydrogels. Upon conducting degradation studies, they observed that a range of degradation rates could be attained. Increased amounts of conjugated methacrylate groups corresponded to increased cross-link densities and decreased degradation rates. However, no significant effect on human aortic endothelial cell cyto-compatibility and proliferation was observed. Rat subcutaneous implants of the GMHA hydrogels showed good biocompatibility, very low inflammatory response, and similar levels of vascularization at the implant edge compared with those of fibrin positive controls. Their results indicate that such GMHA hydrogels can potentially be used in a variety of wound-healing applications. Thus, in general, by systematically tuning the composition and by performing specific chemical modifications, a variety of biocompatible hydrogels can be synthesized and can potentially be used in a wide range of applications such as sustained, targeted drug delivery and tissue engineering.

## 8. Future Perspectives and Concluding Remarks

Novel strategies for the design and synthesis of highly efficient, biocompatible hydrogels will facilitate the creation of new classes of biomaterials for drug delivery and tissue engineering. While most hydrogels are capable of releasing drugs either continuously or intermittently, having accurate, desired release rates would be a major improvement. The use of hydrogel delivery systems for the development of clinically viable products, using recombinant proteins requires further development before these systems can be used without further damaging the proteins. In addition to soft-tissue engineering and drug delivery, bone replacement therapy is another area where hydrogels might find plausible applications. Specific stimuli responsive hydrogels are also a promising group of delivery vehicles in which the release of the encapsulated materials can be controlled by the external stimulus. Such hydrogels may result in significantly superior therapeutic efficacy with minimum side effects. Although the application of hydrogels in gene and vaccine delivery is still relatively scarce, overall, hydrogels have provided a distinctive platform for the development of novel enabling technologies and will continue to do so. 

Hydrogels are extremely crucial therapeutic materials in relation to drug delivery, biosensors and tissue engineering applications due to their biocompatibility, biodegradability to non-toxic products within the body and swelling capacities. More recently, micro- and nanoscale hydrogels have become increasingly popular due of their ability to target areas not accessible to macroscale hydrogels and their use not only in generic drug delivery systems, but also in anticancer therapies, treatments for diabetes, protein delivery systems, biosensor applications, micro-lens development and tissue regeneration. Among synthetic materials, some of the most popular polymeric materials studied in relation to synthesizing hydrogels are poly(*N*-isopropylacrylamide) (pNIPAm), poly(ethylene glycol) (PEG), poly-(ethylene oxide) (PEO), polyvinyl alcohol methylacrylate co-polymers (PVA-MA), polylactic acid (PLA), pluronics and dendrimers. These materials are especially useful in relation to drug delivery, tissue engineering and cancer therapy applications. Glycosaminoglycans such as hyaluronic acid are particularly useful in tissue engineering, while cholesteroyl-bearing pullulans (CHP) has been used in various drug delivery methods. Metal and oxide based materials such as gold nanoparticles and silica-gels are gaining popularity in developing nanoscale drug delivery systems as well. The future holds further advances and developments in and applications for micro- and nanoscale hydrogel systems. 
